# Achieving carbon neutrality in West Africa: The impact of financial development and good governance

**DOI:** 10.1371/journal.pone.0293235

**Published:** 2023-10-26

**Authors:** Justice Gyimah, Ujunwa Angela Nwigwe, Deborah Rubuye Safi, Erica Odwira Opoku, Xilong Yao

**Affiliations:** College of Economics and Management, Taiyuan University of Technology, Taiyuan, China; University of Pitesti, Romania; Institute of Doctoral and Post-doctoral Studies, University Lucian Blaga of Sibiu, ROMANIA

## Abstract

Achieving a net zero carbon has been one of the main agendas for all state and non-state actors. The political system of developing countries sometimes makes both internal and external actors question their efforts toward the agenda. Therefore, this study contributes to previous literature in analyzing the empirical effect of financial development and governance quality on carbon emissions. The study covers sixteen West African countries with data from 1996 to 2021. The study employs the Generalized Method of Moments for the analysis. Financial development in all the models contributes to carbon emissions. However, the effect of governance quality indicators varies depending on the model and the indicator(s) used. Nevertheless, economic governance and political governance in most models contribute to environmental pollution, but institutional governance helps promote environmental quality. Renewable energy and economic growth promote environmental quality through carbon mitigation. However, trade openness promotes environmental pollution by encouraging the release of carbon emissions. Finally, relevant policy implications are proposed based on the empirical findings of the study.

## Introduction

Over the past decades, environmental degradation has become a global concern due to the massive release of carbon dioxide into the environment which is detrimental to life and the ecosystem. This concern has attracted the attention of researchers, environmentalists, and international organizations thus compelling governments and policymakers around the globe to devise new measures for addressing this global menace [1–4]. Alongside this, a lot of work has been done into ensuring carbon neutrality and the various elements needed to achieve the agenda [5]. Since 1992, several policies have been put in place with the aim of mitigating and curbing the effects of carbon dioxide emissions, the 1992 Framework Convention on Climate Change, the 1997 Kyoto Protocol, and the 2016 196-nation Paris Agreement. Additionally, the federal government of the United States established the 101st Federal Climate Policy, which attempts to cut emissions using mitigating gases to lower the rising global temperatures. It is now vital to take action to reduce CO_2_ emissions from the main polluting countries like the west African countries [6].

The ECOWAS region like Nigeria, Liberia, Sierra Leone, Niger, Ghana, etc has set goals to reach a carbon emission peak before 2030 by increasing the proportion of renewable energy in the entire electricity mix by 19% in 2030, as well as accomplishing carbon neutrality [7, 8]. The accomplishment of this might be viewed as an industrial revolution and a crucial turning point in human and African history [9]. In the framework of the current energy system and the political atmosphere, it is still unclear if carbon neutrality is feasible. Hence, this article is justified by four literary threads: (i) the main reasons behind exploring and focusing on West Africa, (ii) focusing on the financial-carbon emissions nexus, (iii) evaluating the role of good governance in increasing environmental protection; and (iv) the effect of good governance on financial development. These concepts are further explained in the remaining paragraphs of this section in relation to the previous literature.

West African countries have commercial quantities of a range of nonrenewable resources, such as wood, oil, and gas. Nigeria for instance is Africa’s top oil producer and one of the top ten oil-exporting economies, and Liberia, Ghana, and Cote d’Ivoire have recently joined the club of oil-rich nations. But for many years, these countries have struggled with economic, financial, and environmental problems. Additionally, emerging West African countries continue to suffer from the "old age, resource curse" syndrome that has affected the majority of African countries [6]. As a result, the expansion of financial inclusion, the rate of population growth, mining operations, agriculture, and industrialization in these countries have all steadily declined over time. Most West African economies are committed to preserving their environmental health while maintaining their financial performance in order to establish a sustainable environment [10].

The connection between financial development and carbon emissions is very important to address. According to numerous studies on the topic, financial development affects carbon emissions both directly and indirectly and the studies indicated financial development has an inevitable role in reducing energy demands. These studies include those by [11–14]. Several studies have been done to provide empirical evidence on the relationship that exists between financial development, energy use, and environmental sustainability and these studies concluded that, financial development is one of the best ways to promote environmental sustainability through energy-efficient equipment use [15, 16]. Even the United Nations (UN) supports climate financing since sizable investments may allow emissions to decline in line with the Paris Agreement. However, [17–19] argue that a strong economy with high financial development pulls foreign investors which mostly becomes a threat to environmental sustainability.

In addition, numerous studies have established and outlined the connection between environmental quality and governance quality [20–22]. The institutions and customs used to carry out the power in the state are generally referred to as governance. The governance controls and seeks to the running of the day-to-day administration of the country [23]. Consequently, the pollution haven hypothesis, which explains why lenient environmental rules in the host country may promote more foreign direct investment from companies looking to avoid costly compliance with regulatory standards in their home nations, is applicable [24, 25]. In this regard, as governments around the world continue to look for ways to encourage sustainable development, the importance of good governance as a crucial tool for accomplishing this objective has recently gained popularity with policymakers and scholars. Governance is believed to have the capability of promoting environmental sustainability when it factors environmental policies into its administration.

After considering all these factors, this study aims to add to the existing literature in three major ways. Previous studies used financial development as one of the independent indicators but failed to address its effect on carbon emissions without governance quality. Therefore, to enrich the previous studies, one more indicator has been added to the financial development, and it has been assessed without the interference of governance quality and its effect on carbon emissions. Secondly, upon thorough literature review, no study has considered this research direction on West Africa as a region. The Panel study in this direction considered a comparative analysis on BRICS, MINT, and G7. However, as a lot of concerns have been raised recently against the governance in most West African countries due to the current happenings in some countries in the region like terrorism, political instability, economic mismanagement, and weak political institutions. In this era of environmental sustainability, it is important to examine how these recent happenings in the region are affecting the fight against global warming. Thirdly, a lot of studies have been done on the effect of the pandemic on energy and energy prices. These studies only focused on the post Covid-19 effect on energy use, energy prices, and carbon emissions in many developing countries. However, since government bodies were challenged during the pandemic, it is important to assess the effect of these government bodies on carbon emissions using energy, economic growth, foreign direct investment, and trade openness as control variables. The study provides empirical evidence of all these raised concerns to enrich the current literature on carbon emissions mitigation.

The rest of the study is organized as follows. section 2 literature review, section 3 elaborates on the method, section 4 presents the results and discusses, and section 5 concludes and renders policy recommendations.

## Literature review

Financial development (FD) has drawn much more attention from academics, researchers, economists, and policymakers and has proven to be a positive factor in economic development [26, 27]. Many studies have found that FD is beneficial to an economy because of the technological dispersion it fosters [28–30]. However, financial progress is a broad notion with many different aspects, it is challenging to quantify with empirical works considering quantitative indicators that are accessible over extended time periods that are the assets to GDP ratio of financial institutions, the GDP ratio of liquid liabilities, and the GDP ratio of deposits [31]. Some literatures [27, 32, 33] detailed FD as a proportion of domestic lending to the private sector to GDP. The conclusions in these studies further estimated that an increase in bank loans to the private sector is thought to contribute to structural transformation and output expansion. It is crucial, nevertheless, how firms employ this resource to be provided through the banks.

There have been several studies on the connection between FD and environmental quality. According to Wudil et al. [34], African continent is considered vulnerable to the risks of climate change due to its poorer financial, scientific, and technology capabilities. The first body of research indicates that by preventing environmental deterioration, FD significantly increases environmental sustainability [31]. According to Tang et al. [35], the relationship between FD and environmental deterioration has been positively associated which results in lowering carbon emissions. To support the argument, the FD in China has also a moderating effect on carbon emissions [36, 37]. Dogan and Seker [38] further examined the link between FD and environmental quality in 23 different countries by applying the FMOLS and DOLS approaches, which concurred with the link between the two variables. According to Lei et al. [39], a country engaging and giving heed to climate finance helps reduce CO_2_, and mitigation funding has a greater impact than adaptation finance. Al Mamun et al. [40] in their study concluded that, green bonds under financial development are the primary drivers of sustainable financing, which considerably reduces CO_2_ over the short and long term.

One vital component of FD and foreign direct investment is that they bring capital, cutting-edge technologies, promoting economic growth and technical improvement and managerial know-how to a country [41]. Multinational firms’ investments also frequently result in the creation of jobs for locals in the host countries. There is a detrimental effect on the environment when the economic activity of the country increases. Additionally, these economic advances depend heavily on the use of energy, particularly fossil fuels. However, some experts contend that financial prosperity may promote technological innovation in green industries but its impact on the environment should be given attention [31, 42]. It is noted that FD supports sustainable regional, national, and international development and tries to create a less polluted atmosphere by offering eco-friendly products. However, Shahbaz et al. [43] found FDI in France as the main cause of environmental pollution. Kivyiro and Arminen [44] also discovered that FDI has a significant impact on the Sub-Saharan region which could be a negative or positive effect. A hospitable FDI environment is equally crucial in the decision-making process of multinational corporations (MNCs) [45]. Foreign direct investment grants strand focuses on the impact of institutions as a subset of governance on it. These institutional systems are laws and beliefs that guide and govern people’s conduct in an economy, which are regarded as national elements [46]. Governance systems and decisions are able to play their role effectively in the economic build-up. As a result, they may be able to influence the laws for MNC operations and foreign investments. Furthermore, property rights, political stability, transparency, and the absence of corruption protect the integrity of local markets [47]. A thriving economy also depends on a consistent set of legal standards. As a result, the function of governance becomes a vital component in boosting a country’s financial development. If a thriving economy is built from its consistent legal standards being set and implemented, then the arm of governance becomes a catalyst element in boosting a country’s financial development. Krifa-Schneider and Matei [48] agreed with this in their research on 33 advanced countries, explaining that political stability coupled with good governance fosters financial development and FDI inflow. One major factor outlined as a barricade to financial development in most countries is corruption [49]. Corruption is viewed as institutional inadequacy and is identified as a key factor influencing FDI flows [50, 51]. Also, corruption is a deterrent that boosts MNCs’ expenditures and deters investors by acting as tools that are detrimental to economic growth through several distorting channels of the economy [52]. Hence, the corruption factor may have an indirect influence on CO_2_ emissions with a direct link to FD [49].

To this end, the review of the salience of governance to sustainable development is prompted by various factors including the present concern about environmental pollution; and weak governance concerns associated with the administration of the policy crisis of environmental pollution. Furthermore, environmental governance rules must be drastically altered to include tangible activities such as supporting environmental projects beyond agreements and summits pertaining to the recent environmental protection needs. In conclusion, while FD can reduce the effects of acute poverty and economic imbalance, and boost a country’s economic growth, it can also have negative effects on environmental quality, hence it is an important issue to address.

## Methodology and data

To assess the impact of financial quality (how well the financial institution manages its resources and how these resources are made available to individuals), governance quality (how government is well organized and responsible in governance dimensions namely government effectiveness, political stability and absence of violence, control of corruption, regulatory quality, voice and accountability, and rule of law) and control variables on carbon emissions from 1996 to 2021, the study follows the studies of Omri et al. [51] and Ofori et al. [46] but employed the empirical model used by Gyimah and Yao [53] thus Generalized Method of Moments (GMM). The model helps to establish a long-term effect without issues of endogeneity and arbitrary heteroscedasticity of any unknown form.

To assess the suitability of the variables, the study first employed the Cross-section dependency test to assess if the variables are interdependent. The study then employed the Panel Unit Root test to assess the stationarity of the variables and the Panel Cointegration test was done. After, these preliminary tests, then the GMM is employed to assess the long-term effect of the independent variables on the dependent variable.

Eq 1 comprises of all the variables for the study thus the effect of financial development indicators, control variables, and governance quality indicators on carbon emissions. The variables are presented in the equation below

$$\begin{array}{l} \ln co_{2_{it}}=\beta_{0}+\beta_{1}\ln e_{it}+\beta_{2}\ln f_{it}+\beta_{3}\ln re_{it}+\beta_{4}\ln t_{it}+\beta_{5}\ln fi_{it}+\beta_{6}\ln dc_{it}+\beta_{7}\ln gc_{it}+\beta_{8}\ln r_{it}+\\ \beta_{9}\ln g_{it}+\beta_{10}\ln v_{it}+\beta_{11}\ln p_{it}+\beta_{12}\ln rl_{it}+\beta_{13}\ln cc_{it}+\varepsilon_{it} \end{array}$$ (1)

In the equation *i* = 1,…,26, t indicates the time from 1996 to 2021 *β*_0_−*β*_14_ are the coefficients, *ε* is the error term.

In order to get the exact impact of governance indexes on carbon emissions, the governance will be grouped into three indicators as economic governance [54], institutional governance [55], and political governance [33]. Therefore, economic governance, dependent variables, control variables, and financial development indicators are presented in Eq 2, institutional governance, dependent variables, control variables, and financial development indicators are presented in Eq 3, and political governance, dependent variables, control variables, and financial development indicators are presented in Eq 4.


$$\begin{array}{l} \ln co_{2_{it}}=\beta_{0}+\beta_{1}\ln e_{it}+\beta_{2}\ln f_{it}+\beta_{3}\ln re_{it}+\beta_{4}\ln t_{it}+\beta_{5}\ln fi_{it}+\beta_{6}\ln dc_{it}+\beta_{7}\ln gc_{it}+\beta_{8}\ln r_{it}+\\ \beta_{9}\ln g_{it}+\varepsilon_{it} \end{array}$$ (2)

$$\begin{array}{l} \ln co_{2_{it}}=\beta_{0}+\beta_{1}\ln e_{it}+\beta_{2}\ln f_{it}+\beta_{3}\ln re_{it}+\beta_{4}\ln t_{it}+\beta_{5}\ln fi_{it}+\beta_{6}\ln dc_{it}+\beta_{7}\ln gc_{it}+\beta_{8}\ln rl_{it}+\\ \beta_{9}\ln cc_{it}+\varepsilon_{it} \end{array}$$ (3)

$$\begin{array}{l} \ln co_{2_{it}}=\beta_{0}+\beta_{1}\ln e_{it}+\beta_{2}\ln f_{it}+\beta_{3}\ln re_{it}+\beta_{4}\ln t_{it}+\beta_{5}\ln fi_{it}+\beta_{6}\ln dc_{it}+\beta_{7}\ln gc_{it}+\beta_{8}\ln v_{it}+\\ \beta_{9}\ln p_{it}+\varepsilon_{it} \end{array}$$ (4)

To further assess the effect of financial indicators and the control variables on carbon emissions without the interference of the governance indicators, Eq 5 is developed. To further assess the effect of governance quality and control variables on carbon emissions without the interference of financial development indicators Eq 6 is developed.


$$\ln co_{2_{it}}=\beta_{0}+\beta_{1}\ln e_{it}+\beta_{2}\ln f_{it}+\beta_{3}\ln re_{it}+\beta_{4}\ln t_{it}+\beta_{5}\ln fi_{it}+\beta_{6}\ln dc_{it}+\beta_{7}\ln gc_{it}+\varepsilon_{it}$$ (5)

$$\begin{array}{l} \ln co_{2_{it}}=\beta_{0}+\beta_{1}\ln e_{it}+\beta_{2}\ln f_{it}+\beta_{3}\ln re_{it}+\beta_{4}\ln t_{it}++\beta_{5}\ln r_{it}+\beta_{6}\ln g_{it}+\beta_{7}\ln v_{it}+\beta_{8}\ln p_{it}+\\ \beta_{9}\ln rl_{it}+\beta_{10}\ln cc_{it}+\varepsilon_{it} \end{array}$$ (6)

### Data

The data of the study is obtained from World Development Indicators (WDI) (https://databank.worldbank.org/source/world-development-indicators), the World Governance Indicators (WGI) (https://databank.worldbank.org/source/worldwide-governance-indicators), and the International Monetary Fund (IMF) (https://www.imf.org/en/Data)and Eviews is the software used for the analysis. The data is from 1996 to 2021 based on the availability of data. The definition and the source of the variables, the statistical description, and the correlation of the data are presented in Tables 1‒3 respectively. The statistical description of the variables and their correlation are important to assess the mean, median, and standard deviation of the data and how each variable is correlated to the other. The correlation results show that Control of corruption and government effectiveness have the strongest positive correlation (0.732) while economic growth and carbon emissions have the lowest positive correlation relationship (0.003). In addition, renewable energy consumption and carbon emissions have the lowest negative correlation (-0.701) while and the highest negative correlation is between political stability and carbon emissions (-0.019). The rest of the results show how each variable correlates with other variables. The data covers all West African countries which have been presented in S1 Appendix.

**Table 1:** The definition and source of the data.

Variable	Indicator	Index	Source
Control of corruption	It is how public power is exercised for private gain	*cc*	WGI
Governance effectiveness	It comprises of the quality of public service, civil service, and freedom from political pressure	*g*	WGI
Political stability	Absence of violence or terrorism	*p*	WGI
Regulatory Quality	The strength of government to implement policies	*r*	WGI
Rule of law	The enforcement of laws and confidence of people in the laws	*rl*	WGI
Voice and accountability	The extent the people are allowed to select their leaders and voice out their grievances	*v*	WGI
Carbon emissions	The release of carbon dioxide into the atmosphere	*co*_2_	WDI
Trade openness	Total of exports and imports	*t*	WDI
Economic growth	Annual GDP per capita	*e*	WDI
Renewable energy	Total renewable energy consumption	*re*	WDI
Domestic credit to private sector	Financial assistance provided to private sector by banks	*dc*	WDI
Foreign Direct Investment	Net inflow of investments	*f*	
Gross capital formation	It captures the fixed assets and net changes within the level of inventories	*gc*	WDI
Financial development	Financial development index	*fi*	IMF

**Table 2:** Statistical description of the data.

	Mean	Median	Max	Min	Std. Dev
ln *co*_2_	-1.324	-1.357	0.119	-2.952	0.738
ln *cc*	3.678	3.758	4.539	1.848	0.472
ln *dc*	2.484	2.548	4.293	-0.303	0.742
ln *e*	0.860	1.053	2.968	-3.427	0.921
ln *f*	0.868	0.942	4.638	-6.089	1.263
ln *fi*	-2.279	-2.309	-1.309	-4.132	0.435
ln *g*	3.441	3.572	4.317	1.342	0.552
ln *gc*	2.984	3.024	4.046	0.092	0.455
ln *p*	3.604	3.868	4.605	1.352	0.764
ln *r*	3.636	3.731	4.401	1.700	0.420
ln *re*	4.153	4.325	4.523	3.034	0.377
ln *rl*	2.628	2.873	4.195	-0.737	1.113
ln *t*	4.058	4.052	4.886	2.794	0.344
ln *v*	3.703	3.761	4.543	2.600	0.425

**Table 3:** Correlation.

	ln *co*_2_	ln *cc*	ln *dc*	ln *e*	ln *f*	ln *fi*	ln *g*	ln *gc*	ln *p*	ln *r*	ln *re*	ln *rl*	ln *t*	ln *v*
ln *co*_2_	1													
ln *cc*	.054	1												
ln *dc*	.590	.350	1											
ln *e*	.003	.035	.098	1										
ln *f*	.219	.037	.209	.179	1									
ln *fi*	.664	.110	.668	.109	.139	1								
ln *g*	.327	.732	.390	-.036	.098	.318	1							
ln *gc*	.461	.164	.557	.044	.282	.430	.323	1						
ln *p*	-.019	.526	.117	.026	.125	-.206	.418	.030	1					
ln *r*	.263	.797	.524	.049	.056	.347	.839	.312	.452	1				
ln *re*	-.701	-.490	-.588	-.055	-.351	-.363	-.522	-.460	-.383	-.475	1			
ln *rl*	.243	.744	.590	.068	.252	.321	.715	.312	.505	.826	-.533	1		
ln *t*	.403	.133	.413	.182	.408	.129	.064	.370	.269	.118	-.500	.180	1	
ln *v*	.159	.632	.379	.070	.082	.198	.603	.184	.503	.619	-.436	.697	.107	1

### Dependent variable

Carbon emissions is the dependent variable for this study. Carbon dioxide is frequently released into the atmosphere by industrial activities, construction, and even agricultural activities. After water vapor, it is the most powerful greenhouse gas released into the atmosphere. Therefore, issues concerning environmental sustainability cannot be addressed without addressing carbon emissions which does not affect only the environment but human existence as well. In this regard, examining the effect of various important indicators on carbon emissions is very important to provide the needed solution to its release and effects.

### Independent variables

The independent variables for the study have been grouped into two; financial development and governance quality;

#### Financial development

Financial development plays a very important role in a country’s development. Following Omri et al. [51] and Ofori et al. [46], the study uses financial development index and domestic credit to private sector as indicators for financial development however after further readings, the study considered gross capital formation to be the third indicator for financial development.

#### Governance quality

The governance system of every country has influence on its environment alongside the well-known implications. In this regard, the study considered the effect of governance quality on carbon emissions by selecting six indicators (voice and accountability, regulation quality, rule of law, control of corruption, political stability, and government effectiveness) to represent it and has been grouped into economic governance, institutional governance, and political governance.

### Control variables

The study uses four different variables as control variables depending on the role they play in the carbon emissions mitigation campaign. These control variables are employed to regulate the effect of the independent variables on carbon emissions. The control variables are; renewable energy, economic growth, trade openness, and foreign direct investment.

### Econometric modeling and procedure

Considering the purpose of the study, Generalized Moment of Methods is proposed to examine the effect of financial development, control variables, and governance quality on carbon emissions in West Africa from 1996 to 2021. The study paid much focus on Omri et al. [51] time series model but the model was transformed to fit the panel data used for the study. Several preliminary tests are conducted to ensure the viability of the data. Firstly, correlation and statistical description are tested, secondly, Panel Unit test root is tested for the stationarity of the variables; thirdly, Cross-sectional dependency test is examined; fourthly, Panel cointegration test is examined; and finally examine the long-term effect. Since the Generalized Method of Moments is capable of checking endogeneity and arbitrary heteroscedasticity of any unknown form, the model is suitable for the analysis.

## Results

The Cross-sectional dependency test was done as part of the study estimation procedure to assess the variables for the study spillover effects. The result of the Cross-sectional dependency test is an indication that any change in each variable of the study could affect that of another country. Based on the significant of the cross-section dependency test results presented in Table 4, there is existence of cross-sectional dependency among the variables. In addition, Panel Unit Root test is performed to examine the order of integration of the study data. The variables are expected to be not stationary at levels and stationary at first difference before the condition of the Panel unit root test can be met. The result is presented in Table 4 which can be concluded that the variables are not stationary at levels but are stationary at first difference therefore the variables are stationary. The study proceeds to assess the cointegration among the variables using Panel cointegration test and the outcomes are presented in Table 5.

**Table 4:** Panel unit root test and cross-sectional dependency test.

	Levin, lin, Chu		Im, Pesaran, Shin		ADF		Cross-sectional Dependency Test
	level	first	level	first	level	first	Breusch-Pagan LM
ln *cc*	-0.114	-5.0678*	0.153	-5.930*	28.929	90.8941*	481.995*
ln *co*_2_	-3.080*	-8.026*	-0.855	-7.850*	43.458***	121.873*	1083.62*
ln *dc*	-3.419*	-6.370*	-0.674	-8.406*	40.154	130.784*	1168.18*
ln *e*	1.064	-6.346*	-1.394***	-5.268*	54.197**	73.2705*	158.0675*
ln *f*	0.676	2.056	-1.939**	-5.728*	50.111**	101.329*	329.927*
ln *fi*	0.038	-8.741*	1.268	-10.80*	19.526	169.149*	714.671*
ln *g*	-2.975*	-7.641*	-1.648**	-8.262*	53.688*	122.442*	525.284*
ln *gc*	-1.274	-10.05*	-0.969	-9.742*	34.505	147.798*	376.325*
ln *p*	-5.231*	-7.290*	-3.556*	-9.234*	75.299*	136.558*	637.952*
ln *r*	-1.660**	-8.516*	-1.211	-9.215*	40.750	143.228*	609.680*
ln *re*	-2.097*	-10.60*	0.422	-8.844*	34.289	139.747*	911.645*
ln *rl*	-1.234	-2.587	-0.897	-5.733*	38.737	90.3414*	449.431*
ln *t*	-0.754	-5.232*	-0.587	-7.680*	29.746	116.487*	385.525*
ln *v*	-2.653*	-11.41*	-3.071*	-11.24*	64.739*	177.226*	322.764

**Table 5:** Panel cointegration test.

	All Variables	ln *fi*	ln *dc*	ln *gc*
All Variables	-2.212*			
ln *r* & ln *g*		2.263*	0.797	1.401**
ln *rl* & ln *cc*		0.447	0.055	0.310
ln *v* & ln *p*		-2.278*	-3.389*	-2.079*
*R* ^2^	0.212			
Adjusted *R*^2^	0.204			
Mean dependent var	0.007			
S.D. dependent var	0.111			

### The long-term empirical analysis

The study employed Generalized Method of Moments to analyze the long-term effect of the independent and control variables on the dependent variable and the results are presented in Table 6. The Generalized Method of Moments is employed because the model is able to deal with endogeneity during estimation. The model is able to check and control all arbitrary heteroscedasticity and endogeneity of any unknown form [53]. Since all the variables are log-transformed, the results of the estimation are regarded as elasticities.

**Table 6:** The results from GMM estimation.

Variable	Coefficient	t-statistics
ln *fi*	0.578*	6.033
ln *dc*	0.098	1.357
ln *gc*	0.009	0.118
ln *r*	0.487*	2.642
ln *g*	0.385*	3.491
ln *rl*	-0.173*	-3.433
ln *cc*	-0.648*	-5.832
ln *v*	0.173***	1.786
ln *p*	-0.078	-1.481
ln *re*	-0.876*	-10.93
ln *e*	-0.064**	-2.134
ln *t*	0.684*	6.867
ln *f*	-0.029	-1.099
*R* ^2^	0.756	
Adjusted *R*^2^	0.741	
Mean dependent var	-1.329	
S.D. dependent var	0.759	

*<1%

**<5%

***<10%

The results presented in Table 6 are based on Eq 1. The equation requires assessing the effect of the independent variables and the control variables on carbon emissions. The results of the study are interesting findings, the results show that only Financial Development Index out of the three Financial Development indicators (Gross Domestic Capita, Financial Development Index, Domestic Credit to Private Sector) has a significant effect on carbon emissions and the effect is positive at 1% significant. The result is contrary to Omri et al. [51] but supported by Ofori et al. [46] although only one Financial Development indicator is significant.

The result further reveals that, Economic Governance reduces environmental quality. Regulation quality and Government effectiveness which are the variables for economic governance has a positive effect on carbon emissions at 1% significant. Regulation quality has 4.9% significant and Government effectiveness has 3.9% significant. However, Institutional Governance helps promote environmental sustainability. The results indicate that at 1% significance, Rule of law and Control of corruption have negative and significant effects on carbon emissions. Rule of law has 1.7% impact on promoting environmental quality while Control of corruption has 6.5% impact on reducing carbon emissions. Only one variable of Political Governance indicators has a significant effect on carbon emissions. Thus, Voice and Accountability has a positive significant effect on carbon emissions at 10% significance, however, Political stability is not significant.

Lastly, the four control variables have shown their effect on carbon emissions although their effects vary. Renewable energy at 1% significance has 8.8% negative and significant effect on carbon emissions. The result is supported by Ofori et al. [46] and the argument made by Gyimah et al. [56] and Justice et al. [57] that, renewable energy helps mitigate carbon emissions and provide reliable grounds for promoting environmental sustainability. Economic growth at 5% significance has 6% negative and significant effect on carbon emissions. Economic growth encourages environmental quality through the mitigation of carbon emissions. However, Trade openness at 1% significance has 6.4% significant and positive effects on carbon emissions. Trade Liberation does not provide a positive effect towards the fight for environmental sustainability and the result is supported by Gyimah et al. [58]. Foreign direct investment has no significant effect on the environment.

### The long-term empirical analysis of governance indicators on financial development-carbon emissions nexus

In order to ascertain the exact effect of governance indicators on environmental sustainability, the governance indicators have been disaggregated into economic governance, institutional governance, and political governance and the results are presented in Table 7. The results show that economic governance promotes environmental quality since it reduces environmental pollution. Regulation quality has a negative and significant effect on carbon emissions but government effectiveness is not significant. Again, institutional governance promotes environmental sustainability thus both variables for institutional governance (rule of law and control of corruption) have negative and significant effects on environmental pollution. Lastly, political governance also helps in the fight against environmental pollution as one of its indicators (political stability) has negative effect on carbon emissions but voice and accountability is not significant. This result slightly differs from the results in Table 6 because, with these results, each indicator stands on its own.

**Table 7:** Long-term effect of governance quality indicators on carbon emissions.

Variables	Economic governance		Institutional governance		Political governance	
	ln *r*	ln *g*	ln *rl*	ln *cc*	ln *v*	ln *p*
ln *fi*	0.756* (8.184)	0.770* (8.470)	0.778* (8.098)	0.658* (7.196)	0.752* (8.136)	0.674* (6.911)
ln *dc*	0.001 (0.011)	-0.025 (-0.357)	0.084 (1.080)	0.077 (1.141)	0.002 (0.027)	0.016 (0.237)
ln *gc*	0.1995* (2.468)	0.182** (2.219)	0.161** (2.073)	0.217* (2.831)	0.198* (2.481)	0.185** (2.361)
ln *r*	-0.049 (-0.569)					
ln *g*		0.027 (0.435)				
ln *rl*			-0.099* (-2.997)			
ln *cc*				-0.255* (-4.006)		
ln *v*					-0.060 (-0.787)	
ln *p*						-0.113* (-2.381)
ln *re*	-0.626* (-8.807)	-0.641* (-9.313)	-0.654* (-9.804)	-0.630* (-9.564)	-0.625* (-8.904)	-0.676* (-9.748)
ln *e*	-0.059*** (-1.749)	-0.058*** (-1.719)	-0.082* (-2.469)	-0.060*** (-1.853)	-0.058*** (-1.721)	-0.060*** (-1.799)
ln *t*	0.660* (6.550)	0.644* (6.394)	0.695* (7.091)	0.735* (7.486)	0.667* (6.575)	0.721* (7.032)
ln *f*	-0.053*** (-1.846)	-0.051*** (-1.783)	-0.033 (-1.195)	-0.063** (-2.275)	-0.052*** (-1.830)	-0.052*** (-1.848)

*<1%

**<5%

***<10%

### Long-term empirical results of financial development and governance quality without each other

Table 8 shows the result of the effect of Financial Development on environmental sustainability without Governance Quality indicators and Table 9 shows the effect of Governance Quality on environmental pollution with Financial Development Indicators. The results in Table 8 show that financial development causes carbon emissions while domestic credit to private sector and gross formation have no effect on carbon emissions. In addition, the results in Table 9 reveal that voice and accountability, regulatory quality, and governance effectiveness contribute to carbon emissions while political stability, control of corruption, and rule of law help mitigate carbon emissions.

**Table 8:** Financial development effect without governance quality on carbon emissions.

Variables	Coefficient	t-statistics
ln *fi*	0.767*	8.478
ln *dc*	-0.017	-0.262
ln *gc*	0.191**	2.407
ln *e*	-0.059***	-1.759
ln *re*	-0.637*	-9.349
ln *f*	-0.052***	-1.803
ln *t*	0.651*	6.554
*R* ^2^	0.652	
Adjusted *R*^2^	0.642	
Mean dependent var	-1.332	
S.D. dependent var	0.757	

*<1%

**<5%

***<10%

**Table 9:** Governance quality without financial development effect on carbon emissions.

Variable	Coefficient	t-statistics
ln *r*	0.850*	4.474
ln *g*	0.405*	3.452
ln *rl*	-0.091***	-1.761
ln *cc*	-0.928*	-8.035
ln *v*	0.222**	2.179
ln *p*	-0.283*	-5.651
Le	-0.040	-1.234
ln *re*	-1.202*	-16.95
ln *f*	-0.038	-1.384
ln *t*	0.749*	8.638
*R* ^2^	0.694	
Adjusted *R*^2^	0.681	
Mean dependent var	-1.297	
S.D. dependent var	0.763	

*<1%

**<5%

***<10%

## Conclusion and policy implications

The main objective of this study is to assess how financial development affects environmental pollution in West Africa in the presence of good governance. Financial development index, gross capital formation, and domestic credit to private sector serve as the three indicators for financial development, and government quality and are grouped into three major categories thus economic governance, institutional governance, and political governance. Renewable energy, economic growth, trade openness, and foreign direct investment are used as the control variables for the study. The data of the study is from 1996 to 2021 from WDI, WGI, and IMF. The Generalized Method of Moments is employed to examine the empirical effects of the study. To assess the environmental impact of the independent variable and the control variable on carbon emissions, there have been six econometric analysis which has summarized the empirical findings into six main strands. The first strand of the empirical findings reveals the relationship between financial development, governance quality, control variables, and carbon emissions. The second strand considers the effect of economic governance and financial development on carbon emissions. The third strand analyzes the impact of institutional governance and financial development on carbon emissions. The fourth strand captures the effect of political governance and financial development on carbon emissions. The fifth strand considers the effect of financial development on carbon emissions without governance quality, and the sixth strand examines the impact of governance quality on carbon emissions without financial development. The results of the study reveals interesting outcomes. The results show that financial development causes environmental pollution throughout all the six strands however, governance quality has different effects on carbon emissions depending on the strand. Nevertheless, from the main Eq (1), economic governance and political governance promote environmental pollution however institutional governance promotes environmental quality. Renewable energy and economic growth encourage environmental quality but trade openness causes harm to the environment by encouraging carbon emissions.

Based on the results of the study, financial development promotes environmental pollution. It is very important for policymakers to take note and implement some financial development strategies in environmental policies to reduce its level of deterioration. The results further reveal that trade openness promotes environmental pollution. Globalization has promoted trade liberation and most countries within Africa are suffering from its bad effects despite the economic impact of globalization. Policymakers should put restrictions on trade to control the trade of high-carbon technologies. The results show that institutional governance reduces carbon emissions. Therefore, various government bodies are encouraged to promote the rule of law and control corruption. These factors give the people hope to embrace the fight against carbon emissions. As explained by the United Nations Environment Programme (UNEP) that for the past 60 year almost 40% of internal conflict can be associated with natural resources and further explained how the exploitation of these natural resources damages the environment. Rule of law would create conducive environment for the people to support environmental sustainability.

## Supporting information

S1 Appendix (DOCX)
