# Supplementary material for: Achieving carbon neutrality in West Africa: The impact of financial development and good governance
Source: PLoS One. 2023 Oct 26;18(10):e0293235. doi: 10.1371/journal.pone.0293235 (PMC10602346; doi:10.1371/journal.pone.0293235)
Supplement: S1 Appendix — (DOCX) [file pone.0293235.s001.docx]

**Appendix**

Table 10 The Sixteen West African Countries for the Study

| West Africa Sixteen Countries | | | |
| --- | --- | --- | --- |
| [Benin](http://pitt.libguides.com/aecontent.php?pid=346196&sid=3536902) | [Gambia](http://pitt.libguides.com/aecontent.php?pid=346196&sid=3536934) | [Liberia](http://pitt.libguides.com/aecontent.php?pid=346196&sid=3536974) | [Nigeria](http://pitt.libguides.com/aecontent.php?pid=346196&sid=3537012) |
| [Burkina Faso](http://pitt.libguides.com/aecontent.php?pid=346196&sid=3536353) | [Ghana](http://pitt.libguides.com/aecontent.php?pid=346196&sid=3536936) | [Mali](http://pitt.libguides.com/aecontent.php?pid=346196&sid=3536977) | [Senegal](http://pitt.libguides.com/aecontent.php?pid=346196&sid=3537044) |
| [Cape Verde](http://pitt.libguides.com/aecontent.php?pid=346196&sid=3536352) | [Guinea](http://pitt.libguides.com/aecontent.php?pid=346196&sid=3536951) | [Mauritania](http://pitt.libguides.com/aecontent.php?pid=346196&sid=3536996) | [Sierra Leone](http://pitt.libguides.com/aecontent.php?pid=346196&sid=3501738) |
| [Côte D'Ivoire](http://pitt.libguides.com/aecontent.php?pid=346196&sid=3536343) | [Guinea-Bissau](http://pitt.libguides.com/aecontent.php?pid=346196&sid=3536955) | [Niger](http://pitt.libguides.com/aecontent.php?pid=346196&sid=3537003) | [Togo](http://pitt.libguides.com/aecontent.php?pid=346196&sid=3980834) |
